# Advanced imaging in adult diffusely infiltrating low-grade gliomas

**DOI:** 10.1186/s13244-019-0793-8

**Published:** 2019-12-18

**Authors:** Nail Bulakbaşı, Yahya Paksoy

**Affiliations:** 1 Medical Faculty, University of Kyrenia, Sehit Yahya Bakır Street, Karakum, Mersin-10 Kyrenia, Turkish Republic of Northern Cyprus Turkey; 2 Selcuk University, Konya, Turkey

**Keywords:** Astrocytoma (grade II), Oligodendroglioma (adult), Magnetic resonance imaging

## Abstract

The adult diffusely infiltrating low-grade gliomas (LGGs) are typically IDH mutant and slow-growing gliomas having moderately increased cellularity generally without mitosis, necrosis, and microvascular proliferation. Supra-total resection of LGG significantly increases the overall survival by delaying malignant transformation compared with a simple debulking so accurate MR diagnosis is crucial for treatment planning. Data from meta-analysis support the addition of diffusion and perfusion-weighted MR imaging and MR spectroscopy in the diagnosis of suspected LGG. Typically, LGG has lower cellularity (ADC_min_), angiogenesis (rCBV_max_), capillary permeability (*K*_trans_), and mitotic activity (Cho/Cr ratio) compared to high-grade glioma. The identification of 2-hydroxyglutarate by MR spectroscopy can reflect the IDH status of the tumor. The initial low ADC_min_, high rCBV_max_, and *K*_trans_ values are consistent with the poor prognosis. The gradual increase in intratumoral Cho/Cr ratio and rCBV_max_ values are well correlated with tumor progression. Besides MR-based technical artifacts, which are minimized by the voxel-based assessment of data obtained by histogram analysis, the problems derived from the diversity and the analysis of imaging data should be solved by using artificial intelligence techniques. The quantitative multiparametric MR imaging of LGG can either improve the diagnostic accuracy of their differential diagnosis or assess their prognosis.

## Key points

- LGG has lower cellularity (ADC_min_), angiogenesis (rCBV_max_), capillary permeability (*K*_trans_), and mitotic activity (Cho/Cr) than HGG.
- Initial low ADC_min_ and high CBV_max_ and *K*_trans_ values are consistent with the poor prognosis.
- A gradual increase in Cho/Cr ratio and rCBV_max_ values is well-correlated with tumor progression.
- Critical distortions in quantifying parameters can be minimized by proper ROI selection and voxel-based assessment.
- Quantitative multiparametric MRI can either improve the diagnostic accuracy of conventional MRI or provide a better assessment.

## Introduction

According to the 2016 update of World Health Organization (WHO) on the classification of tumors of the central nervous system, diffusely infiltrating low-grade gliomas (LGG) in the adult include the WHO grade II astrocytoma and oligodendrogliomas but rarely oligoastrocytomas [1, 2]. Because most of the oligoastrocytomas have genetic profiles typical of either diffuse astrocytoma or oligodendroglioma and the new WHO classification discourages the diagnosis of tumors as oligoastrocytoma or mixed glioma [1, 2]. Typically, LGGs are slow-growing tumors having moderately increased cellularity without prominent mitosis, necrosis, and microvascular proliferation [1]. So they usually have more indolent clinical course than high-grade gliomas (HGG), included anaplastic astrocytoma (grade III), anaplastic oligodendroglioma (grade III), and glioblastoma (grade IV). Their incidence peaks are at an earlier age of life (third to fourth decades), as opposed to those of HGG (sixth to seventh decades) [1, 3]. The gliomas show diffuse infiltration of adjacent and distant brain structures that are largely irrespective of its histological grade and even its low grade, it usually shows microscopic peritumoral white matter tract invasion [1]. The demonstration of this diffuse infiltration is very important for the accurate glioma diagnosis.

The precise diagnosis of LGG is critical to make an appropriate treatment decision, because the supra-total resection (defined as the removal of a margin around the tumor visible on FLAIR images) significantly increases the overall survival and delays the malignant transformation of LGG [4, 5]. The surgical biopsy is reserved in markedly diffuse lesions like a gliomatosis cerebri pattern [4]. Because of the lack of consensus among various diagnosis, management, and treatment options in LGG, the joint tumor section of the American Association of Neurological Surgeons and the Congress of Neurological Surgeons published the evidence-based guidelines in 2015 [6]. These include a systematic review as well as an evidence-based clinical practice guideline about the role of imaging in the management of adult diffusely infiltrating LGG [7].

Magnetic resonance imaging (MRI) is the modality of choice in the diagnostic assessment of LGG and provides a reasonably good delineation of the gliomas [5, 7]. The quantitative assessment of advanced MR imaging techniques has long been used both for the preoperative evaluation of gliomas by providing molecular and metabolic information in addition to the routine anatomical evaluation [7, 8]. There is also a significant role in follow-up particularly in the differentiation of the post-irradiation changes and of the pseudo-progression [7]. The quantitative multiparametric MRI approach can improve the diagnostic accuracy of conventional MRI [8]. In this educational review, we aim to define the spectrum and diagnostic value of the available advanced imaging techniques in neuro-oncology. We will also review the added value as well as the possible pitfalls of using quantitative MRI techniques in addition to conventional MRI. Finally, the review will assess the importance of advanced MRI acquisition technique standardization in clinical practice of neuro-oncology.

## Imaging technique

The multiparametric MRI evaluation of LGG includes the conventional anatomical MRI sequences, namely T2, fluid-attenuated inversion recovery (FLAIR) and pre- and post-contrast T1-weighted images. In addition, the protocol includes advanced MRI techniques such as susceptibility-weighted imaging (SWI), diffusion-weighted imaging (DWI), perfusion-weighted imaging (PWI), MRI spectroscopy (MRS), and functional MRI (fMRI) techniques. The T1 and FLAIR sequences should be preferred as volumetric acquisitions particularly for the follow-up to make an appropriate comparison about the tumor progression or the high-grade transformation of LGG [7]. The subtracted images of pre- and post-contrast 3D T1-weighted or FLAIR images provide a better assessment of tumoral enhancement. The SWI is quite important to identify the intratumoral calcifications, microbleeds, vasculature and allows the calculation of intratumoral susceptibility score (ITSS) [9]. The DWI and the calculation of apparent diffusion coefficient (ADC) should always be in the routine diagnostic protocol, but at present diffusion kurtosis imaging is usually reserved for research purposes. Dynamic susceptibility contrast (DSC) and/or dynamic contrast-enhanced (DCE) perfusion imaging techniques are crucial for both the initial diagnosis and the follow-up of LGG [7, 9, 10]. Arterial spin labeling (ASL) perfusion is an alternative perfusion technique, which uses the magnetically labeled water protons as a contrast material and can also be used for the same purposes but it does not allow the calculation of relative cerebral blood volume (rCBV) [11]. It is usually preferred in patients with previous severe allergic/anaphylactoid reaction to a gadolinium-based contrast agent; patients with severe renal disease (eGFR < 30 mL/min/1.73 m^2^) or acutely deteriorating renal function; patients who would be at risk of nephrogenic systemic fibrosis; and patients who are, or might be, pregnant [11, 12]. The MRS is usually reserved for diagnostic verification and problem solving but not for the routine diagnosis [7]. But recently, high-resolution MRS technique with selective TE and different editing method is being used in glioma diagnosis to detect 2-hydroxyglutarate (2-HG), which accumulates within the gliomas having isocitrate dehydrogenase 1 and 2 (IDH 1/2) mutations [13, 14]. Diffusion tensor imaging and tractography with or without fMRI are usually reserved for preoperative evaluation of selected cases. The new emerging MRI techniques, such as amide proton transfer (APT) imaging [15, 16], sodium MRI [17], and MR elastography [18] can also be used for glioma grading but they are usually reserved for research purposes.

## Radiomic data for the histopathological features of LGGs

According to guideline, conventional MRI is the first-order technique (Level II evidence) to identify the location of the tumor and its relation to adjacent cerebral structures [7]. The diffusion and perfusion images are quite helpful in the assessment of LGG (Level II, Class II/III evidence) by providing a better identification of tumoral heterogeneity than anatomic MRI sequences and provide the differential diagnosis for tumor subtype and grade [7]. The diagnostic potential of MRS and positron emission tomography (Level III, Class III evidence) are still being defined [7]. The data of radiologic-pathologic correlation from meta-analysis of current literature [10, 11, 15, 19–31] demonstrates that the tumoral cellularity correlates with low T2 signal and low ADC; atypia with low ADC and high fractional anisotropy; mitotic activity with high Cho/Cr, Cho/NAA ratios, rCBV, and APT signal; the microvascular proliferation with high rCBV, volume transfer coefficient (*K*_trans_), volume fraction of plasma (*V*_p_), and ITSS; and necrosis with necrotic cavity and high lactate levels on MR spectroscopy.

The differential diagnosis and grading of gliomas are still debating. The quantitative multiparametric MR imaging approach can better differentiate LGG from HGG with very high sensitivity (84.2%), and specificity (100%) than conventional MRI, thereby reducing the risk of inappropriate or delayed surgery, respectively [8]. By this purpose, a significant amount of quantitative data has been collected during the last decade and a lot of different cut-off values have been defined for the grading of gliomas in the literature as summarized in Table 1 [7, 9–11, 19–30]. The most common parameters defined in literature for grading are maximum relative cerebral blood volume (rCBV_max_), minimum normalized ADC (nADC_min_), and choline to creatine (Cho/Cr) ratio. The defined cut-off values for glioma grading are 1.75 for rCBV_max_ in the largest cohort [19] ranging between 0.94 and 3.34 [9–11, 19–22], 1.07 × 10^−3^ mm^2^/s for nADC_min_ in the largest cohort [24] ranging between 0.31 and 1.31 [23–28] and 1.56 for Cho/Cr ratio in the largest cohort [19] ranging between 1.3 and 2.04 [29–31]. There was a considerable variation in cut-off values because of the difference in the study design, types of MRI devices, coils, sequences, post-processing algorithms, picking up hot points vs. histogram analysis, and diverse methodology in determining cut-off values. A standardized, multicenter acquisition and analysis protocol for the imaging data is feasible and highly reproducible, having a comparable high diagnostic accuracy in glioma grading according to the new WHO 2016 classification scheme [21].

**Table 1:** Radiomic data for differential diagnosis of low-grade vs high-grade gliomas

Parameters	Low-grade glioma	High-grade glioma	Cut of value [Ref] (range) [Ref]
rCBV_max_	Low	High	1.76 [10, 19] (0.94–3.34) [9–11, 19–22]
rCBV_MD_	Low	High	1.44 [22] (1.08–1.81) [22]
nADC_min_	High	Low	1.07 × 10^−3^ mm^2^/s [24] (0.31–1.31) [23–28]
Cho/Cr ratio	Low	High	1.56 [19] (1.3–2.04) [29–31]
MK_MD_	Low	High	0.17 [28] (0.11–0.28) [28]
FA_TC_	Low	High	0.3 [25] (0.14–0.63) [25]
MD_min_	High	Low	0.98 mm^2^/s [25] (0.76–0.91) [25]
*k*_trans_	High	Low	1.18 [22] (0.91–1.45) [22]
*V*_e_	High	Low	1.43 [22] (1.06–1.80) [22]
ITTS grade	1.2	2.6	NA [9]
APT signal (%)	Low	High	2.23% [15] (1.53%–3.70%) [15]

*rCBV*_*max*_ maximum relative cerebral blood volume, *rCBV*_*MD*_ standardized mean difference of rCBV_max,_ *nADC*_*min*_ normalized minimum apparent diffusion coefficient, *Cho/Cr* choline/creatine, *MK*_*MD*_ mean difference in mean kurtosis, *FA*_*TC*_ odds ratio of fractional anisotropy in the tumor core, *MD*_*min*_ minimum mean diffusivity, *k*_*trans*_ standardized mean difference of volume transfer coefficient, *V*_*e*_ standardized mean difference of volume fraction of extravascular extracellular space, *ITTS* intratumoral susceptibility score, *APT* percent amide proton transfer signal

Typically, LGG has lower cellular density than HGG so has a higher ADC_min_ and minimum mean diffusivity, and lower fractional anisotropy and mean kurtosis values than HGG [23–28]. The optimal threshold was 0.98 × 10^−3^ mm^2^/s for ADC_min_ and 0.17 for the mean difference in mean kurtosis [26, 28]. The LGG has lower perfusion parameters such as *K*_trans_, volume fraction of extravascular extracellular space (*V*_e_), mean vascular density, and rCBV_max_ values than HGG [9, 10, 19, 22]. This is primarily due to the fact that they are less prone to secret vascular endothelial growth factor, having low microvascular proliferation and lack of immature, hyperpermeable neo-microvascularity [9, 10, 19, 22]. The rCBV_max_ threshold of 1.76 has the highest diagnostic accuracy for diffuse astrocytoma [10]. The LGG has also a lower maximum mean relative tumor blood flow/normal white matter ratio [11] and relatively lower ITSS degrees [9] than HGG, primarily due to a lack of tortuous, disorganized, dilated, and leaky tumoral capillaries. The LGG has significantly lower Cho/Cr or Cho/ N-acetyl aspartate (NAA) ratios and higher myoinositol to Cr ratio than HGG due to having lower membrane turnover rates and production of proteolytic enzymes [29, 30]. LGG has a significantly lower APT signal intensity than HGGs [15, 16]. There is not a meaningful change in protein content of tumor and a moderate correlation between APT signal intensity and Ki-67 proliferation index [14]. Histogram analysis of APT imaging provides increased accuracy for the identification of contrast-enhancing LGG that mimics HGG [16].

Perfusion and diffusion images may also play a role (Level III) in consideration of tumor prognosis and in distinguishing different classes of LGG in terms of prognosis [7]. Poor outcome is well correlated with low ADC_min_ values ranging between 0.799 × 10^−3^ mm^2^/s and 1.69 × 10^−3^ mm^2^/s [32–34], high rCBV_max_ values ranging between 1.46 and 5.195 [32, 33, 35] and high permeability with *K*_trans_ values more than 0.05 min^−1^ and *V*_p_ values more than 5 ml/100 g [36].

In determining high-grade transformation, anatomical MRI (level II) is the first-order technique [7]. New contrast enhancement and increase in tumor size more than 3 mm per year may signify a transformation to a higher grade [7, 37]. Serial PWI and MRS (level III) are also useful for astrocytic tumors, baseline, and gradual longitudinal elevations in rCBV_max_ values and Cho/Cr ratios are associated with shorter time to tumor progression [7]. However, these can be difficult to standardize in clinical practice for oligodendrogliomas and mixed gliomas. Higher growth rate more than 3 mm per year [37], higher baseline rCBV_max_ values more than 1.52–1.75 [37–39] and higher Cho/Cr ratio more than 2.4 [37] are well correlated with high-grade transformation. In transforming LGG, a significant increase in rCBV_max_ can occur up to 12 months before contrast enhancement becomes apparent on T1-weighted MR images [39] and a significant increase in Cho/Cr ratio can occur up to 15 months before the rCBV_max_ elevation [37].

## Radiomic data for the genetic and molecular features of LGGs

According to the updated WHO 2016 classification, 75–80% of grade II diffusely infiltrating astrocytomas have IDH1/2 mutation and 20–25% do not [1, 3]. An IDH1/2 mutant diffusely infiltrating astrocytoma has also had a loss of nuclear alpha-thalassemia/mental retardation syndrome X-linked expression (ATRX) status and TP53 mutations [1, 3]. Although WHO 2016 is based on the basis of combined phenotypic and genotypic classification as well as the generation of integrated diagnoses, WHO grading of gliomas remained unchanged [1]. Essentially LGG has mild nuclear atypia, moderate pleomorphism, high degree of cellular differentiation, and low MIB-1, with intrinsic capacity to progress to IDH1 mutant anaplastic astrocytoma or glioblastoma [1, 3].

There is a big survival difference between IDH1-mutated vs. wild-type gliomas. Although IDH1 mutant glioblastoma has still significantly worse outcomes than grade II and III gliomas, there are no differences in survival between IDH mutant grades II and III [40]. For this reason, Shirahata et al. proposed a novel, improved grading system for IDH-mutant astrocytic gliomas [41]. The premise is cyclin-dependent kinase Inhibitor 2A/B homozygous deletion with combination of necrosis and copy number variation has the most relevant results for survival but the proliferation (mitotic count) has only a minor influence on survival [41].

**Fig. 1:**
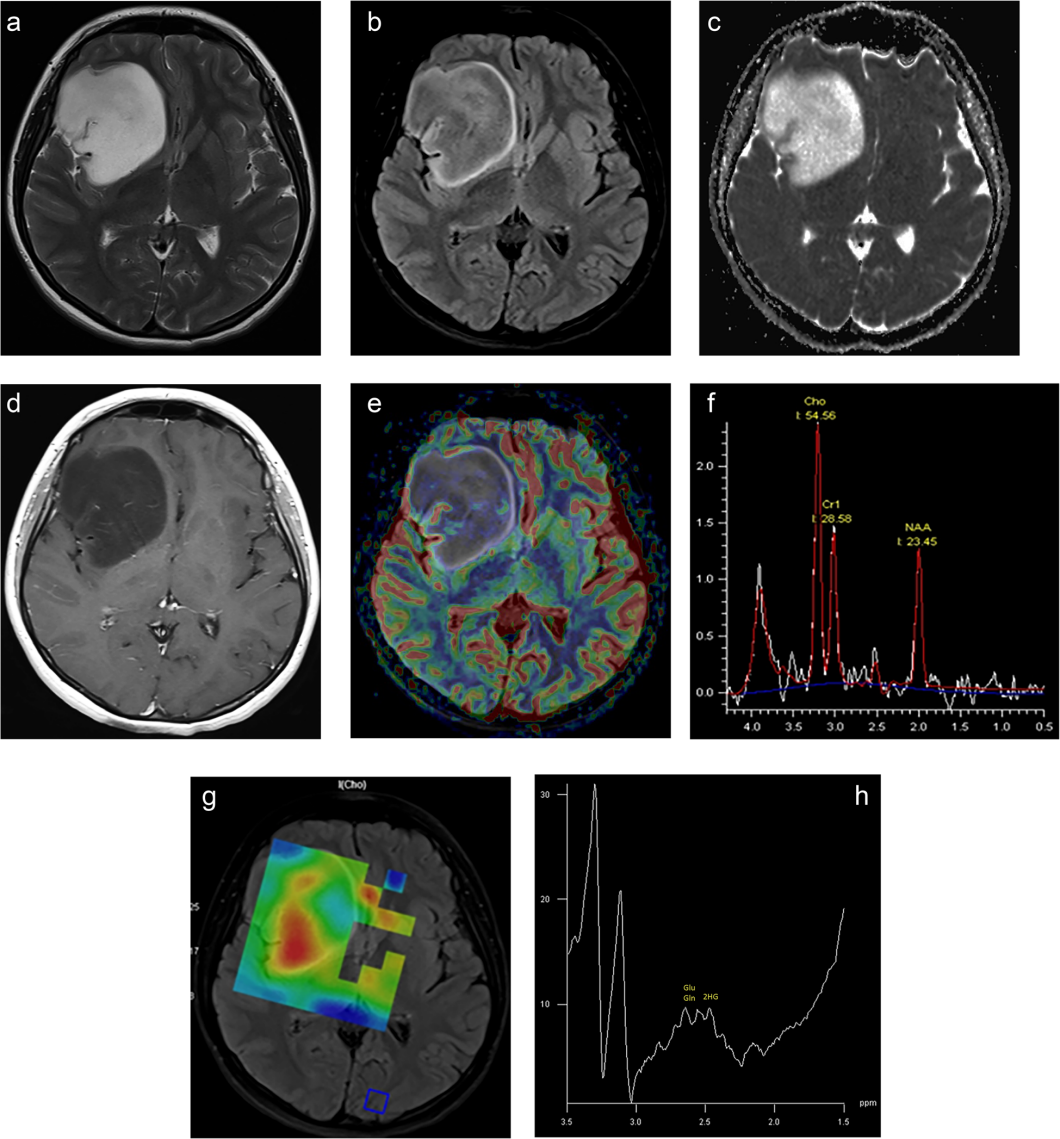
Grade II astrocytoma with IDH1 mutation, 30% p53, and 3% Ki67 is located on the posterior aspect of the right frontal lobe on axial MR images. There is a mismatch sign on T2 (**a**) and FLAIR (**b**) images. The tumor has a high signal on ADC (**c**) images and does not enhance on post-contrast T1-weighted image (**d**). The tumor has low rCBV_max_ values compared to normal parenchyma on the DSC-perfusion image (**e**). Increased Cho/Cr ratio inside the tumor borders is prominent on both the MR spectrum obtained by 144 ms echo time (**f**) and colored Cho map (**g**). The high-resolution MR spectroscopy by 69 ms of echo time (**h**) reveals the 2-HG peak causing a triplet within the glutamine-glutamate complex (Glu-Gln), which is consistent with the existence of IDH1 mutation

The IDH mutant LGG is usually located in the frontal lobe followed by temporal lobe and infratentorial location (Fig. 1). They are mostly solid and do not enhance. They have usually well-defined border and T2/FLAIR “mismatch” sign represented as homogeneous high signal on T2 sequence but bright rim and dark center on FLAIR images [42]. Typically, they have higher ADC_min_ and lower rCBV_max_ values than wild-type tumors and are represented with a slight increase in Cho/NAA as well as Cho/Cr ratios [10, 42, 43]. Radiomic features extracted from the optimal texture analysis of ADC and T2 FLAIR images play an important role in the noninvasive prediction of the IDH1 mutation and loss of ATRX expression status in LGGs [43]. Choi and colleagues showed the existence of 2-HG and glutamate multiplets in patients with IDH-mutated grade II-III tumors, with 100% sensitivity and specificity [13].

**Fig. 2:**
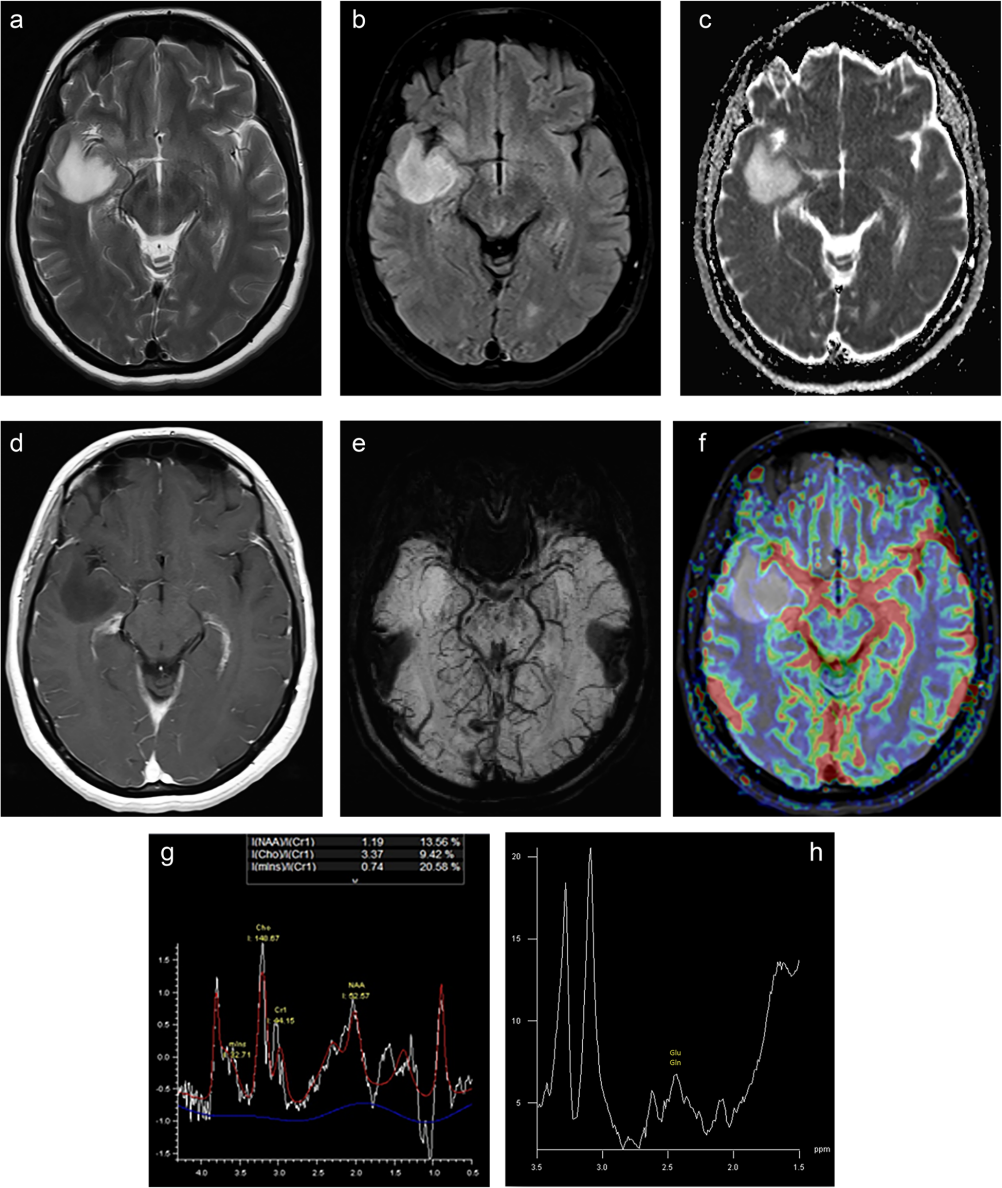
Astrocytoma, grade II with the absence of IDH1 mutation (IDH wild-type), loss of ATRX expression, 20% p53, and 3.3% Ki67 is located on the right temporal lobe on axial MR images. The tumor has a high signal on T2 (**a**), FLAIR (**b**), and ADC (**c**) images and faint enhancement on post-contrast T1-weighted image (**d**). The tumor has low ITSS on SW image (**e**) and rCBV_max_ values compared to normal parenchyma on the DSC-perfusion image (**f**). The tumor has increased Cho/Cr ratio and low NAA/Cr ratio on the MR spectrum obtained by 144 ms echo time (**g**). The high-resolution MR spectroscopy by 69 ms of echo time (**h**) reveals a singlet due to the glutamine-glutamate complex (Glu-Gln), which is consistent with the absence of 2-HG peak and IDH1 mutation

IDH-wild-type LGGs may not have been demonstrated because imaging and histopathology features look low grade, but molecular and clinical features suggest an early stage of primary glioblastoma [44]. Recent literature recommends the use of diffuse astrocytic glioma, IDH-wild-type, with molecular features of glioblastoma, WHO grade IV a.k.a. “Molecular GBM” diagnosis [45]. This is basically based on the existence of epidermal growth factor amplification or combined whole chromosome 7 gain, whole chromosome 10 loss (+ 7/− 10) or telomerase reverse transcriptase promoter mutation, regardless of its histological WHO grade [45]. IDH wild-type diffuse astrocytomas are more likely to exhibit contrast enhancement with intratumoral necrosis and peritumoral edema, but not cyst (Fig. 2). The ADC_mean_ of 1.2 can be used as an optimal cutoff value to differentiate IDH wild-type and IDH-mutant gliomas irrespective of WHO grade and tumors with ADC_mean_ less than 1.08 had poor survival [46]. The absence of a 2-HG peak in MR spectroscopy is also consistent with the IDH wild-type tumors [13, 14]. Furthermore, recent literature also demonstrates that IDH-mutated astrocytomas have higher ADC [46–48] and lower rCBV values [47–49], an APT signal [15, 16], the relaxation-weighted sodium signal to total sodium signal ratio [17], oxygen extraction fraction [50], and tumor stiffness [18] than IDH wild-type tumors.

**Fig. 3:**
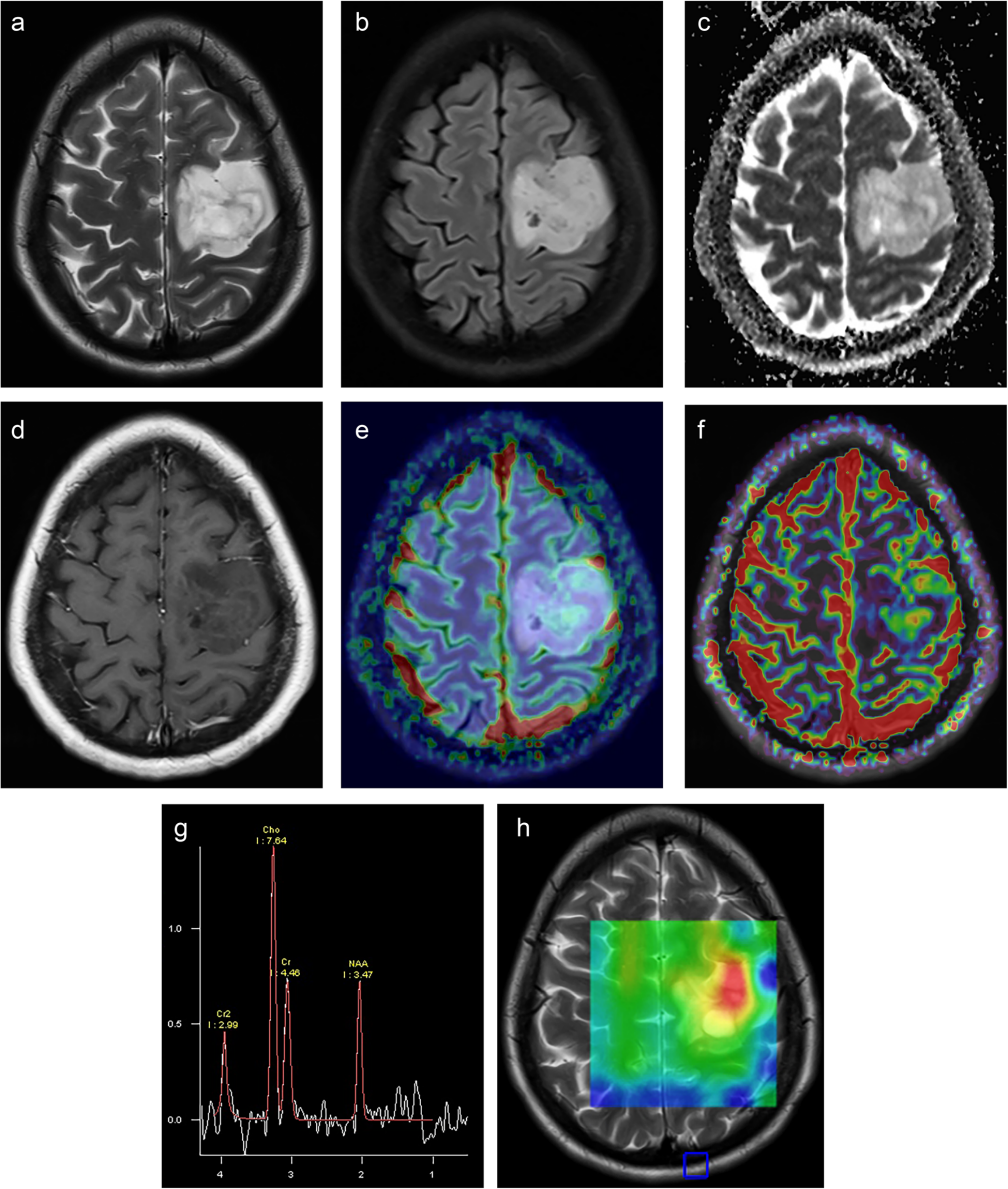
Oligodendroglioma, grade II with 1p19q-codeletion, intact ATRX expression, immune-negative p53, and 2% Ki67 is located on the premotor area of the left frontal lobe on axial MR images. The irregularly contoured tumor has a heterogeneous high signal on T2 (**a**), FLAIR (**b**), and ADC (**c**) images and has a central intratumoral small low signal area consistent with calcification/hemorrhage. It has faint enhancement on post-contrast T1-weighted image (**d**). The tumor has relatively higher rCBV_max_ values than normal parenchyma on DSC-perfusion image (**e**) and *k*_trans_ values on DCE-perfusion image (**f**). The tumor has also high Cho/Cr ratio on the MR spectrum obtained by 144 ms echo time (**g**) at the similar areas of increased perfusion on colored Cho map (**h**)

According to WHO 2016 update, diffusely infiltrating oligodendroglioma (Fig. 3) is a slow-growing glioma with IDH1 or IDH2 mutation and codeletion of chromosomal arms 1p and 19q (1p19q-codeletion) [1]. They have higher rCBV and *V*_e_ and lower ADC_mean_ values than the same grade diffusely infiltrating astrocytomas because of having a chicken wire-fine capillary network causing higher tissue perfusion even in grade II [51–57]. Higher *V*_e_ values are usually with the presence of cortical involvement and calcification and/or hemorrhage [53]. The rCBV ratio, greater than 1.6 is predictive of the 1p19q-codeleted genotype with 92% sensitivity and 76% specificity [54]. Cho/Cr ratio had the highest predictive value, with moderate accuracy (69%) when combined with rCBV_max_ [55]. They have also higher ITSS than the same grade diffuse astrocytomas due to increased angiogenesis, dense vascularity, microbleeds, or microcalcifications [52].

## Possible pitfalls and solutions

**Fig. 4:**
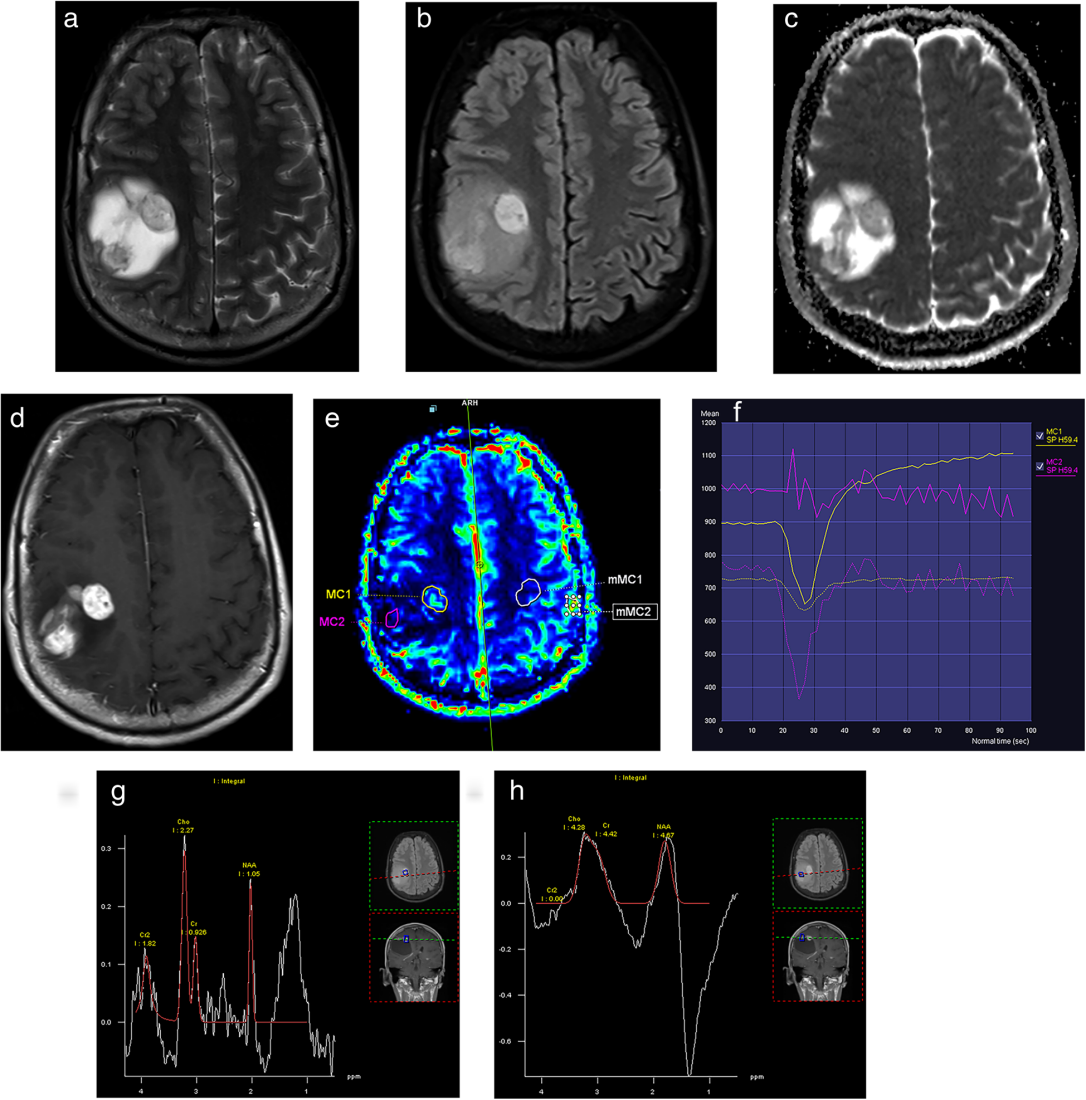
Pilocytic astrocytoma, grade I with immune-negative IHD-1, CD138 and Neu N, and 1% Ki67 is located on the right fronto-parietal lobe on axial MR images. The lobulated but regularly contoured tumor has cystic and solid components on T2 (**a**) and FLAIR (**b**) images and relatively low ADC values (**c**). The solid components of the tumor have heterogenous enhancement on post-contrast T1-weighted image (**d**). On DSC-perfusion image (**e**) two ROIs picked areas showing strong (MC1) and weak (MC2) enhancement and their mirrored counterpart areas (mMC1 and mMC2) from normal parenchyma. The perfusion signal to time graph (**f**) shows increased perfusion curve on MC1 tumoral core (solid yellow curve) compared to mMC1 normal parenchyma curve (dotted yellow curve) but distorted irregular curve on MC2 weakly enhanced area (pink solid curve) due to partial volume effect of cystic component of tumor causing erroneous rCBV values. Similarly, while MR spectrum obtained by 144 ms echo time from MC1 region (**g**) is consistent with tumoral spectrum with increased Cho/Cr ratio and decreased NAA, the MR spectra from MC2 region (**h**) show distorted and unrecognizable curves due to the same reason

The first problem preventing a proper data quantification are MRI artifacts, which are mainly related to magnetic field like susceptibility or inhomogeneity artifacts, or to patient caused by (in)voluntary motion, blood flow, or cerebrospinal fluid pulsation [58, 59]. The magnetic field-based distortions can be reduced by using less sensitive pulse sequences, shorter TE values, wider receiver bandwidths or by applying parallel imaging techniques, and patient-based artifacts can be eliminated by proper immobilization and gating techniques [58, 59]. Small tumor diameter and vascular structures, hemorrhagic, cystic/necrotic, and calcific changes in the tumoral area can also cause critical distortions in quantifying parameters. So the selection of a proper region of interest (ROI) avoiding this kind of areas or the use of histogram analysis can minimize these distortive effects (Fig. 4). The usage of histogram analysis, compared with the use of the current hotspot technique, can increase the diagnostic accuracy and the interobserver reproducibility in glioma grading as well as potentially improve patient care [56, 57]. Also emerging AI algorithms can provide the voxel-based assessment of data obtained by histogram analysis or other methods. The voxel-based assessment of imaging data provides new quantitative information, which is invisible to human assessment and can more precisely extract and use thousands of different and new radiomic features, which are validated as the quantitative imaging biomarkers to characterize intratumoral dynamics throughout diagnosis and treatment [60–63]. The second problem is the analysis of data. Recent multiparametric MRI techniques produce a significant amount of imaging data with massive diversity from patient to patient. The analysis and post-processing of this large volume of data is not only complex and time-consuming but also lacks standardization. The newly emerging AI techniques using diagnostic hypotheses and scalable machine-learning algorithms have the potential of automated processing of large data volumes and can enhance the current performance of quantitative cancer imaging [63]. Providing standardization is more difficult because of the variety of hardware and software produced by different vendors, mostly makes the exact comparison of the results difficult. Also, the differences in the scanner type, magnetic field strength, acquisition parameters, protocols, and determination of standard threshold levels make this comparison less reliable. The standardized, multicenter acquisition and analysis protocols can generate more feasible and highly reproducible data and increase the diagnostic accuracy [21]. The AI algorithms using the data mined by machine-learning methods can help to minimize the effects caused by lack of standardization resulting in more reliable results.

## Conclusion

The parameters obtained from the quantification of multiparametric brain MRI can provide an adequate diagnosis of LGG and help to differentiate them from HGG. The LGG has a lower cellularity (ADC_min_), angiogenesis (rCBV_max_), capillary permeability (*K*_trans_), and mitotic activity (Cho/Cr) than HGG. Besides these basic parameters, thousands of different and new radiomic features have been defined in the literature and more will continue to emerge with the advent of AI techniques. The initial low ADC_min_ values, high rCBV_max_, and *K*_trans_ values are consistent with the poor prognosis. The gradual increase in intratumoral Cho/Cr ratio and rCBV_max_ values are well correlated with tumor progression. The major problems in the quantitative multiparametric MR imaging of LGGs include the diversity of imaging equipment and techniques. This can be minimized by using a comparable standardized, multicenter acquisition and analysis protocols and the analysis of large volume data, which can be solved by the automated processing methods of AI. The radiomic features obtained by quantitative multiparametric MRI can enhance the current performance and the clinical potential of a predictive cancer diagnosis.

## Data Availability

The datasets used and/or analyzed during the current study are available from the corresponding author on reasonable request.

## Abbreviations

2-HG: 2-hydroxy-glutarate
ADC: Apparent diffusion coefficient
AI: Artificial intelligence
APT: Amide proton transfer
ASL: Arterial spin labeling
Cho: Choline
Cr: Creatine
DCE: Dynamic contrast-enhanced
DSC: Dynamic susceptibility contrast
DWI: Diffusion-weighted imaging
FLAIR: Fluid-attenuated inversion recovery
fMRI: Functional MRI
HGG: High-grade glioma
IDH: Isocitrate dehydrogenase
ITSS: Intratumoral susceptibility score
*K*_trans_: Volume transfer coefficient
LGG: Low-grade glioma
MRI: Magnetic resonance imaging
MRS: MRI spectroscopy
NAA: n-acetyl aspartate
PWI: Perfusion-weighted imaging
rCBV: Relative cerebral blood volume
SWI: Susceptibility-weighted imaging
*V*_e_: Volume fraction of extravascular extracellular space
*V*_p_: Volume fraction of plasma
WHO: World Health Organization
