# Correction to: Advanced imaging in adult diffusely infiltrating low-grade gliomas

**DOI:** 10.1186/s13244-020-00862-x

**Published:** 2020-04-22

**Authors:** Nail Bulakbaşı, Yahya Paksoy

**Affiliations:** 1 Medical Faculty, University of Kyrenia, Sehit Yahya Bakır Street, Karakum, Mersin-10, Kyrenia, Turkish Republic of Northern Cyprus Turkey; 2 Selcuk University, Konya, Turkey

**Correction to: Insights Imaging**

**https://doi.org/10.1186/s13244-019-0793-8**

The original article "Advanced imaging in adult diffusely infiltrating low-grade gliomas" contains errors in Table 1 in rows k_trans_ and V_e_; the correct version of Table 1 can be viewed in this Correction article.

**Table 1:** Radiomic data for differential diagnosis of low-grade vs high-grade gliomas

Parameters	Low-grade glioma	High-grade glioma	Cut of value^Ref^ (Range)^Ref^
rCBV_max_	Low	High	1.76 [10, 19] (0.94–3.34) [9–11, 19–21]
rCBV_MD_	Low	High	1.44 [22] (1.08–1.81) [22]
nADC_min_	High	Low	1.07 × 10^−3^ mm^2^/s [24] (0.31–1.31) [23–28]
Cho/Cr ratio	Low	High	1.56 [19] (1.3–2.04) [29–31]
MK_MD_	Low	High	0.17 [28] (0.11–0.28) [28]
FA_TC_	Low	High	0.3 [25] (0.14–0.63) [25]
MD_min_	High	Low	0.98 mm^2^/s [25] (0.76–0.91) [25]
k_trans_	Low	High	1.18 [22] (0.91–1.45) [22]
V_e_	Low	High	1.43 [22] (1.06–1.80) [22]
ITTS grade	1.2	2.6	NA [9]
APT signal (%)	Low	High	2.23% [15] (1.53%–3.70%) [15]

*rCBV*_*max*_ maximum relative cerebral blood volume, *rCBV*_*MD*_ standardized mean difference of rCBV_max,_*nADC*_*min*_ Normalized minimum apparent diffusion coefficient, *Cho/Cr* Choline/Creatine, *MK*_*MD*_ mean difference in mean kurtosis, *FA*_*TC*_ Odds ratio of fractional anisotropy in the tumor core, *MD*_*min*_ Minimum mean diffusivity, *k*_*trans*_ standardized mean difference of volume transfer coefficient, *V*_*e*_ standardized mean difference of volume fraction of extravascular extracellular space, *ITTS* Intratumoral susceptibility score, *APT* Percent amide proton transfer signal

## References

[1] Louis DN, Ohgaki H, Wiestler OD, Cavenee WK (2016) WHO classification oftumours of the central nervous system, revised 4th edition. IARC, Lyon.

[2] Sahm F, Reuss D, Koelsche C et al (2014) Farewell to oligoastrocytoma: insitu molecular genetics favor classification as either oligodendroglioma orastrocytoma. Acta Neuropathol 128(4):551–559. 10.1007/s00401-014-1326-7 10.1007/s00401-014-1326-7 25143301

[3] Bready D, Placantonakis DG (2019) Molecular pathogenesis of low-gradeglioma. Neurosurg Clin N Am 30(1):17–25. 10.1016/j.nec.2018.08.011 10.1016/j.nec.2018.08.011 PMC6260953 30470401

[4] Duffau H (2016) Long-term outcomes after supratotal resection of diffuselow-grade gliomas: a consecutive series with 11-year follow-up. ActaNeurochir (Wien) 158(1):51–58. 10.1007/s00701-015-2621-3 10.1007/s00701-015-2621-3 26530708

[5] National Comprehensive Cancer Network. Central nervous system cancers (Version 1.2019). https://www.nccn.org/professionals/physician_gls/pdf/cns.pdf. Accessed Mar 5 2019 10.6004/jnccn.2015.0148 26483059

[6] Olson JJ, Kalkanis SN, Ryken TC (2015). Evidence-based clinical practiceparameter guidelines for the treatment of adults with diffuse low-gradeglioma: introduction and methods. J Neurooncol.

[7] Fouke SJ, Benzinger T, Gibson D, Ryken TC, Kalkanis SN, Olson JJ (2015) Therole of imaging in the management of adults with diffuse low-gradeglioma: a systematic review and evidence-based clinical practice guideline. JNeurooncol 125(3):457–479. 10.1007/s11060-015-1908-9 10.1007/s11060-015-1908-9 26530262

[8] Caulo M, Panara V, Tortora D et al (2014) Data-driven grading of braingliomas: a multiparametric MR imaging study. Radiology 272(2):494–503. 10.1148/radiol.14132040. 10.1148/radiol.14132040 24661247

[9] Li X, Zhu Y, Kang H (2015). Glioma grading by microvascularpermeability parameters derived from dynamic contrast-enhanced MRI andintratumoral susceptibility signal on susceptibility weighted imaging. CancerImaging.

[10] Delgado AF, Delgado AF (2017). Discrimination between glioma grades IIand III using dynamic susceptibility perfusion MRI: a meta-analysis. AJNR AmJ Neuroradiol.

[11] Kong L, Chen H, Yang Y, Chen L (2017) A meta-analysis of arterial spinlabelling perfusion values for the prediction of glioma grade. Clin Radiol72(3):255–261. 10.1016/j.crad.2016.10.016. 10.1016/j.crad.2016.10.016 27932251

[12] ACR Committee on Drugs and Contrast Media (2018) ACR manual oncontrast media (Version 10.3, 2018). https://www.acr.org/-/media/ACR/Files/Clinical-Resources/Contrast_Media.pdf. Accessed 2018.

[13] Choi C, Ganji SK, DeBerardinis RJ (2012). 2-hydroxyglutarate detectionby magnetic resonance spectroscopy in IDH-mutated patients with gliomas. Nat Med.

[14] Tietze A, Choi C, Mickey B (2018). Noninvasive assessment of isocitratedehydrogenase mutation status in cerebral gliomas by magnetic resonancespectroscopy in a clinical setting. J Neurosurg.

[15] Suh CH, Park JE, Jung SC, Choi CG, Kim SJ, Kim HS (2019). Amide protontransfer-weighted MRI in distinguishing high- and low-grade gliomas: asystematic review and meta-analysis. Neuroradiology.

[16] Park JE, Kim HS, Park KJ, Choi CG, Kim SJ (2015) Histogram analysis of amideproton transfer imaging to identify contrast-enhancing low-grade braintumor that mimics high-grade tumor: increased accuracy of MR perfusion.Radiology 277(1):151–161. 10.1148/radiol.2015142347. 10.1148/radiol.2015142347 25910226

[17] Biller A, Badde S, Nagel A (2016). Improved brain tumor classification bysodium MR imaging: prediction of IDH mutation status and tumor progression.AJNR. Am J Neuroradiol.

[18] Pepin KM, McGee KP, Arani A (2018). MR elastography analysis ofglioma stiffness and IDH1-mutation status. AJNR Am J Neuroradiol.

[19] Law M, Yang S, Wang H (2003). Glioma grading: sensitivity, specificity, and predictive values of perfusion MR imaging and proton MRspectroscopic imaging compared with conventional MR imaging. AJNR AmJ Neuroradiol.

[20] Bulakbasi N, Kocaoglu M, Farzaliyev A, Tayfun C, Ucoz T, Somuncu I (2005). Assessment of diagnostic accuracy of perfusion MR imaging in primary andmetastatic solitary malignant brain tumors. AJNR Am J Neuroradiol.

[21] Anzalone N, Castellano A, Cadioli M (2018). Brain gliomas: multicenterstandardized assessment of dynamic contrast-enhanced and dynamicsusceptibility contrast MR images. Radiology.

[22] Liang J, Liu D, Gao P (2018). Diagnostic values of DCE-MRI and DSC-MRIfor differentiation between high-grade and low-grade gliomas: acomprehensive meta-analysis. Acad Radiol.

[23] Bulakbasi N, Guvenc I, Onguru O, Erdogan E, Tayfun C, Ucoz T (2004) Theadded value of the apparent diffusion coefficient calculation to magneticresonance imaging in the differentiation and grading of malignant braintumors. J Comput Assist Tomogr 28(6):735–46 10.1097/00004728-200411000-00003 15538145

[24] Server A, Kulle B, Gadmar ØB, Josefsen R, Kumar T, Nakstad PH (2011) Measurements of diagnostic examination performance using quantitativeapparent diffusion coefficient and proton MR spectroscopic imaging in thepreoperative evaluation of tumor grade in cerebral gliomas. Eur J Radiol 80(2):462–70. 10.1016/j.ejrad.2010.07.017 10.1016/j.ejrad.2010.07.017 20708868

[25] Miloushev VZ, Chow DS, Filippi CG (2015) Meta-analysis of diffusion metricsfor the prediction of tumor grade in gliomas. AJNR Am J Neuroradiol 36(2):302–8. 10.3174/ajnr.A4097 10.3174/ajnr.A4097 PMC7965672 25190201

[26] Zhang L, Min Z, Tang M, Chen S, Lei X, Zhang X (2017) The utility ofdiffusion MRI with quantitative ADC measurements for differentiating high-grade from low-grade cerebral gliomas: evidence from a meta-analysis. JNeurol Sci 373:9–15. 10.1016/j.jns.2016.12.008Erratum in: JNeurol Sci. 2017;375:103–6

[27] Liang R, Wang X, Li M et al. (2014) Potential role of fractionalanisotropy derived from diffusion tensor imaging in differentiatinghigh-grade gliomas from low-grade gliomas: a meta-analysis. Int J ClinExp Med 7(10):3647–53 PMC4238464 25419413

[28] Falk Delgado A, Nilsson M, van Westen D, Falk Delgado A (2018). Gliomagrade discrimination with MR diffusion kurtosis imaging: a meta-analysis ofdiagnostic accuracy. Radiology.

[29] Castillo M, Smith JK, Kwock L (2000). Correlation of myo-inositol levels andgrading of cerebral astrocytomas. AJNR Am J Neuroradiol.

[30] Wang Q, Zhang H, Zhang J (2016). The diagnostic performance ofmagnetic resonance spectroscopy in differentiating high-from low-gradegliomas: a systematic review and meta-analysis. Eur Radiol.

[31] Usinskiene J, Ulyte A, Bjørnerud A (2016). Optimal differentiation ofhigh- and low-grade glioma and metastasis: a meta-analysis of perfusion, diffusion, and spectroscopy metrics. Neuroradiology.

[32] Hilario A, Sepulveda JM, Perez-Nuñez A (2014). A prognostic modelbased on preoperative MRI predicts overall survival in patients with diffusegliomas. AJNR Am J Neuroradiol.

[33] Cuccarini V, Erbetta A, Farinotti M (2016). Advanced MRI maycomplement histological diagnosis of lower grade gliomas and help inpredicting survival. J Neurooncol.

[34] Villanueva-Meyer JE, Wood MD, Choi BS (2018). MRI features and IDHmutational status of grade II diffuse gliomas: impact on diagnosis andprognosis. AJR Am J Roentgenol.

[35] Law M, Oh S, Babb JS (2006). Low-grade gliomas: dynamicsusceptibility-weighted contrast-enhanced perfusion MR imaging-predictionof patient clinical response. Radiology..

[36] Nguyen TB, Cron GO, Mercier JF et al. (2015) Preoperative prognostic valueof dynamic contrast-enhanced MRI-derived contrast transfer coefficient andplasma volume in patients with cerebral gliomas. AJNR Am J Neuroradiol36(1):63–69. 10.3174/ajnr.A4006 10.3174/ajnr.A4006 PMC7965903 24948500

[37] Hlaihel C, Guilloton L, Guyotat J, Streichenberger N, Honnorat J, Cotton F (2010). Predictive value of multimodality MRI using conventional, perfusion, and spectroscopy MR in anaplastic transformation of low-gradeoligodendrogliomas. J Neurooncol.

[38] Law M, Young RJ, Babb JS et al. (2008) Gliomas: predicting time toprogression or survival with cerebral blood volume measurements atdynamic susceptibility-weighted contrast-enhanced perfusion MR imaging.Radiology 247(2):490–98. 10.1148/radiol.2472070898 10.1148/radiol.2472070898 PMC3774106 18349315

[39] Danchaivijitr N, Waldman AD, Tozer DJ (2008). Low-grade gliomas: dochanges in rCBV measurements at longitudinal perfusion-weighted MRimaging predict malignant transformation?. Radiology.

[40] Back M, Jayamanne DT, Brazier D (2019). Influence of molecularclassification in anaplastic glioma for determining outcome and futureapproach to management. J Med Imaging Radiat Oncol.

[41] Shirahata M, Ono T, Stichel D (2018). Novel, improved gradingsystem(s) for IDH-mutant astrocytic gliomas. Acta Neuropathol.

[42] Patel SH, Poisson LM, Brat DJ (2017). T2-FLAIR mismatch, an imagingbiomarker for IDH and 1p/19q status in lower-grade gliomas: a TCGA/TCIA project. Clin Cancer Res.

[43] Ren Y, Zhang X, Rui W (2019). Noninvasive prediction of IDH1 mutationand ATRX expression loss in low-grade gliomas using multiparametric MRradiomic features. J Magn Reson Imaging.

[44] Hasselblatt M, Jaber M, Reuss D (2018). Diffuse astrocytoma, IDH-wildtype: a dissolving diagnosis. J Neuropathol Exp Neurol.

[45] BratDJ, AldapeK, ColmanH, etal. (2018) cIMPACT-NOWupdate3:recommended diagnostic criteria for "diffuse astrocytic glioma, IDH-wildtype, with molecular features of glioblastoma, WHO grade IV. Acta Neuropathol 136(5):805–10. 10.1007/s00401-018-1913-0 10.1007/s00401-018-1913-0 PMC6204285 30259105

[46] Wu CC, Jain R, Radmanesh A et al. (2018) Predicting genotype and survivalin glioma using standard clinical MR imaging apparent diffusion coefficientimages: a pilot study from the cancer genome atlas. AJNR Am J Neuroradiol 39(10):1814–20. 10.3174/ajnr.A5794 10.3174/ajnr.A5794 PMC7410731 30190259

[47] Smits M, van den Bent MJ (2017). Imaging correlates of adult gliomagenotypes. Radiology.

[48] Kickingereder P, Sahm F, Radbruch A et al. (2015) IDH mutation status isassociated with a distinct hypoxia/angiogenesis transcriptome signaturewhich is non-invasively predictable with rCBV imaging in human glioma. SciRep 5:16238. 10.1038/srep16238 10.1038/srep16238 PMC4633672 26538165

[49] Leu K, Ott GA, Lai A (2017). Perfusion and diffusion MRI signatures inhistologic and genetic subtypes of WHO grade II-III diffuse gliomas. JNeurooncol.

[50] Stadlbauer A, Zimmermann M, Kitzwögerer M (2017). MR imaging-derived oxygen metabolism and neovascularization characterization forgrading and IDH gene mutation detection of gliomas. Radiology.

[51] Bian W, Khayal IS, Lupo JM (2009). Multiparametric characterization ofgrade 2 glioma subtypes using magnetic resonance spectroscopic, perfusion, and diffusion imaging. Transl Oncol.

[52] Lin Y, Xing Z, She D (2017). IDH mutant and 1p/19q co-deletedoligodendrogliomas: tumor grade stratification using diffusion-, susceptibility-, and perfusion-weighted MRI. Neuroradiology.

[53] Yoon HJ, Ahn KJ, Lee S (2017). Differential diagnosis of oligodendroglialand astrocytic tumors using imaging results: the added value of perfusionMR imaging. Neuroradiology.

[54] Jenkinson MD, Smith TS, Joyce KA et al. (2006) Cerebral blood volume, genotype and chemosensitivity in oligodendroglial tumours.Neuroradiology 48(10):703–13. 10.1007/s00234-006-0122-z PMC1592467 16937145

[55] Chawla S, Krejza J, Vossough A (2013). Differentiation betweenoligodendroglioma genotypes using dynamic susceptibility contrastperfusion-weighted imaging and proton MR spectroscopy. AJNR Am JNeuroradiol.

[56] Emblem KE, Scheie D, Due-Tonnessen P (2008). Histogram analysis ofMR imaging-derived cerebral blood volume maps: combined gliomagrading and identification of low-grade oligodendroglial subtypes. AJNRAm J Neuroradiol.

[57] Emblem KE, Nedregaard B, Nome T (2008). Glioma grading by usinghistogram analysis of blood volume heterogeneity from MR-derivedcerebral blood volume maps. Radiology.

[58] Dietrich O, Reiser MF, Schoenberg SO (2008) Artifacts in 3T MRI: physicalbackground and reduction strategies. Eur J Radiol 65(1):29–35 10.1016/j.ejrad.2007.11.005 18162353

[59] Vargas MI, Delavelle J, Kohler R, Becker CD, Lovblad K (2009). Brain and spineMRI artifacts at 3Tesla. J Neuroradiol.

[60] Kang Y, Choi SH, Kim YJ (2011). Gliomas: histogram analysis of apparentdiffusion coefficient maps with standard- or high-b-value diffusion-weighted MR imaging--correlation with tumor grade. Radiology.

[61] Zhou M, Scott J, Chaudhury B et al. (2018) Radiomics in brain tumor: imageassessment, quantitative feature descriptors, and machine-learningapproaches. AJNR Am J Neuroradiol 39(2):208–16. 10.3174/ajnr.A5391 10.3174/ajnr.A5391 PMC5812810 28982791

[62] Zhang X, Yan LF, Hu YC (2017). Optimizing a machine learning basedglioma grading system using multi-parametric MRI histogram and texturefeatures. Oncotarget.

[63] Lee MH, Kim J, Kim ST et al. (2019) Prediction of IDH1 mutation in GBMusing machine learning technique based on quantitative radiomic data.World Neurosurg 125:e688–e696. 10.1016/j.wneu.2019.01.157 10.1016/j.wneu.2019.01.157 30735871

